# Nuclear Import of β-Dystroglycan Is Facilitated by Ezrin-Mediated Cytoskeleton Reorganization

**DOI:** 10.1371/journal.pone.0090629

**Published:** 2014-03-05

**Authors:** Alejandra Vásquez-Limeta, Kylie M. Wagstaff, Arturo Ortega, Dorothy H. Crouch, David A. Jans, Bulmaro Cisneros

**Affiliations:** 1 Departamento de Genética y Biología Molecular, Centro de Investigación y Estudios Avanzados del Instituto Politécnico Nacional (CINVESTAV-IPN), México D.F., Mexico; 2 Nuclear Signalling Laboratory, Department of Biochemistry and Molecular Biology, Monash University, Clayton, Victoria, Australia; 3 School of Dentistry, University of Dundee, Dundee, Scotland, United Kingdom; University of Birmingham, United Kingdom

## Abstract

The β-dystroglycan (β-DG) protein has the ability to target to multiple sites in eukaryotic cells, being a member of diverse protein assemblies including the transmembranal dystrophin-associated complex, and a nuclear envelope-localised complex that contains emerin and lamins A/C and B1. We noted that the importin α2/β1-recognised nuclear localization signal (NLS) of β-DG is also a binding site for the cytoskeletal-interacting protein ezrin, and set out to determine whether ezrin binding might modulate β-DG nuclear translocation for the first time. Unexpectedly, we found that ezrin enhances rather than inhibits β-DG nuclear translocation in C2C12 myoblasts. Both overexpression of a phosphomimetic activated ezrin variant (Ez-T567D) and activation of endogenous ezrin through stimulation of the Rho pathway resulted in both formation of actin-rich surface protrusions and significantly increased nuclear translocation of β-DG as shown by quantitative microscopy and subcellular fractionation/Western analysis. In contrast, overexpression of a nonphosphorylatable inactive ezrin variant (Ez-T567A) or inhibition of Rho signaling, decreased nuclear translocation of β-DG concomitant with a lack of cell surface protrusions. Further, a role for the actin cytoskeleton in ezrin enhancement of β-DG nuclear translocation was implicated by the observation that an ezrin variant lacking its actin-binding domain failed to enhance nuclear translocation of β-DG, while disruption of the actin cytoskeleton led to a reduction in β-DG nuclear localization. Finally, we show that ezrin-mediated cytoskeletal reorganization enhances nuclear translocation of the cytoplasmic but not the transmembranal fraction of β-DG. This is the first study showing that cytoskeleton reorganization can modulate nuclear translocation of β-DG, with the implication that β-DG can respond to cytoskeleton-driven changes in cell morphology by translocating from the cytoplasm to the nucleus to orchestrate nuclear processes in response to the functional requirements of the cell.

## Introduction

Dystroglycan (DG), an essential component of the dystrophin associated protein complex (DAPC), is an integral plasma membrane receptor composed of two subunits, α and β, which link the cytoskeleton to the extracellular matrix. α-DG, the extracellular peripheral subunit binds laminin and other lamin G (LG) module-containing extracellular matrix proteins via its extensively glycosylated mucin-like region. β-DG, a type 1 transmembrane glycoprotein, binds to the carboxy-terminal domain of α-DG via its extracellular face, as well as to a number of actin binding proteins on its intracellular face, thus connecting DG to the actin cytoskeleton under various circumstances [1].

Besides its structural role at the plasma membrane, β-DG has emerged as a multifunctional platform for adhesion and adhesion-mediated signaling in various cell types and tissues. There are many interacting partners which associate with the cytoplasmic domain of β-DG to modulate different cellular functions, including cytoskeleton remodeling via its interaction with ezrin [2], [3], the Extracellular signal-related kinase-Mitogen-activated protein (ERK-MAP) kinase cascade [4], and in concert with integrins, the dynamics and assembly of cellular adhesions in myoblasts, thereby modulating myoblast anchorage, and migration [5], [6], the latter process being critically regulated by Src-mediated phosphorylation of β-DG at tyrosine 890 [7].

Interestingly, β-DG has been localized in the nucleus of different cell lines [8]-[10], and it has recently been revealed that β-DG is imported into the nucleus, through recognition of a nuclear localization signal (NLS), located in its juxtamembrane region, which is recognized by the importin (IMP)α/β heterodimer, a member of the cellular nuclear transport machinery [11], [12]. These unexpected findings expand the possible functional properties of β-DG beyond its known role as a transmembrane adhesion/signaling protein. Previously we have shown that β-DG can enter the nucleus to interact with different nuclear envelope (NE) proteins [13], including lamin A/C, lamin B1 and emerin, enabling it to modulate nuclear envelope structure and function in myoblasts.

**Figure 1:**
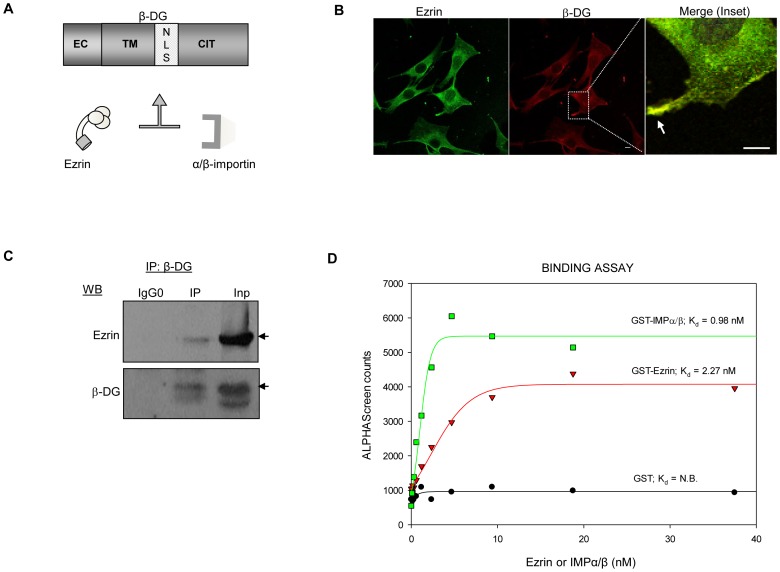
β-DG interacts with ezrin in C2C12 myoblasts. **A.** Schematic of the domain structure of β-DG. EC, extracellular domain; TM, transmembrane domain; NLS, nuclear localization signal; CYT, cytoplasmic tail. The NLS of β-DG serves as binding site for both importin α2/β1 and ezrin. **B.** C2C12 cells grown on glass coverslips were fixed and double stained with anti-ezrin (green) and anti- β-DG antibodies prior to be analyzed by confocal laser scanning microscopy (CLSM). Merged images show colocalization between ezrin and β-DG (yellow), specifically at plasma membrane projections (Inset); scale bar is 10 µm. **C.** C2C12 total extracts were immunoprecipitated (IP) using an anti-β-DG antibody or control antibody (IgG0), and precipitated proteins subjected to SDS-PAGE/Western analysis employing anti-β-DG or anti-ezrin antibodies. **D.** The IMPα2/β1 heterodimer interacts with the NLS of β-DG with higher affinity than ezrin. Increasing concentrations (0-60 nm) of GST-tagged ezrin, GST-tagged predimerized IMPα2/β1 or GST alone as a control were incubated with a GFP-β-DGNLS fusion protein and an ALPHA-Screen assay performed as described in Materials and Methods. Sigmoidal curves were fitted using the SigmaPlot software to determine the apparent dissociation constants (Kd) as indicated. Each data point represents the average of three measurements from a single typical experiment from a series of three separate experiments.

Distribution of β-DG in different subcelular compartments, including to the plasma membrane and within the nucleus, implies that trafficking of β-DG may be tightly regulated to attain a critical concentration of the protein in each location in response to specific stimuli. Nuclear transport of proteins is regulated at multiple levels via a diverse range of mechanisms, such as modulation of the accessibility of the target signal by the IMP receptor proteins. In particular, intra- or intermolecular masking of NLSs within cargo proteins to prevent IMP recognition is one of the most common mechanisms utilised to regulate the efficiency of nuclear transport (reviewed in [14], [15]). Intermolecular masking occurs when binding of a heterologous protein prevents the NLS-IMP interaction. Interestingly, the same basic residues belonging to the NLS of β-DG where shown to mediate the interaction with the cytoskeleton adaptor protein ezrin [2], suggesting that this interaction may also modulate access of the β-DG NLS to IMPs and thereby nuclear translocation (Fig. 1A).

In this study we demonstrate that nuclear import of β-DG is not restrained by ezrin; instead, we find that cytoskeletal reorganization mediated by ezrin activation enhances the nuclear trafficking of β-DG through the IMPα/β nuclear import pathway.

## Materials and Methods

### Cell culture, drug treatment and transfection

Mouse C2C12 myoblast cell line was used in all the experiments. C2C12 cells were grown in Dulbecco’s modified Eagle’s Medium (DMEM) (Invitrogen, Carlsbad, CA, USA) supplemented with 10% (v/v) fetal bovine serum, 50 U/ml penicillin, 50 µg/ml streptomycin, and 1 mM sodium pyruvate at 37°C in a humidified 5% CO_2_ atmosphere_._ Where appropriate, cells were grown in serum-free DMEM for 6 h prior to incubation with 1 µg/ml of L-α-lysophosphatidic acid (Sigma-Aldrich, St Louis, Missouri, USA) in supplemented DMEM for 1, 2 or 30 min at 37°C, or incubation with 1µg/ml cell permeable C3-transferase (Rho inhibitor; Cytoskeleton Inc. Denver, CO. USA) in supplemented DMEM for 12 h. To induce actin cytoskeleton disorganization, C2C12 cells were treated when appropriate with 6 µM cytochalasin B (Sigma-Aldrich, St Louis, Missouri, USA) in supplemented DMEM for 1 h. C2C12 myoblasts were transfected using Lipofectamine 2000 (Invitrogen, Carlsbad, California, USA), following the manufacturer’s protocol. Briefly, for indirect immunofluorescence assays 2 µg plasmid DNA in 100 µl serum-free DMEM medium was mixed with 4 µl Lipofectamine 2000 and incubated for 30 min at room temperature. The transfection mixture was then combined with 2 ml fresh medium (37°C) and added to cells grown overnight on coverslips (15×15 mm) at 75% confluence. For western blotting experiments, cells seeded on 60 mm wells were transfected using 4 µg of plasmid DNA and 8 µl Lipofectamine 2000, as described above. After 24 h, cells were harvested prior to subcellular fractionation and western blotting analysis. Where indicated, stable transfection was performed by incubating transfected cells in DMEM containing 200 µg/ml puromycin for 8 days.

### Antibodies

The following primary antibodies were used: rabbit polyclonal antibodies against β-DG (JAF) [16], ezrin (H276), Sp3 (D-20), calnexin (H70) (Santa Cruz Biotechnology, Santa Cruz, CA., USA), and IMPβ1 (3E9) (Abcam, Cambridge, UK); rabbit monoclonal anti-phospho-ezrin (Thr567)/radixin (Thr564)/moesin (Thr558) antibody (41A3) (Cell Signalling Technology, Inc. Danvers, MA. USA); goat polyclonal (C20) and mouse monoclonal (7D11) anti-β-DG antibodies (Santa Cruz Biotechnology, Santa Cruz, CA., USA).

### Plasmid constructs

The eukaryotic expression vector GFP-ezrin encoding full-length ezrin fused to GFP was previously described [17]. Mutant derivatives of ezrin fused to GFP, Ez-T567D, Ez-T567A and Ez-567EDΔNLS, were generated by site-directed mutagenesis using GFP-ezrin as the template, a high fidelity polymerase *Pfu* turbo, (Invitrogen, Carlsbad, CA, USA) and the following oligonucleotides: forward and reverse primer for Ez-T567D were 5′CCGGGACAAGTACAAGGACCTGCGGCAGATCCGG 3′ and 5′CCGGATCTGCCGCAGGTCCTTGTACTTGTCCCGG 3′ respectively; for Ez-T567A, 5′CCGGGACAAGTACAAGGCGCTGCGGCAGATCCGG 3′, and 5′ CCGGATCTGCCGCAGCGCCTTGTACTTGTCCCGG 3′; for Ez-ET567DΔNLS, 5′ GCCCTCCTGGAAGAGGCGGAGGATGAAGTTGAAGAG 3′ and 5′ CTCTTCAACTTCATCCTCCGCCTCTTCCAGGAGGGC 3′. To generate a vector encoding an ezrin variant lacking the actin-binding domain fused to GFP (EzΔABD), the amino acids 1-470 were amplified by PCR from the GFP-ezrin template, using the following oligonucleotides: forward 5′ CGCCAAGAATTCCCGAATGCCGAAACCAATC 3′, and reverse 5′ CGGTTAGTCGACGAGGGTGCTGTCATCACCAGGTG 3′, flanked by *Eco*RI and *SalI* sites respectively, and cloned in frame into plasmid pEGFP-N1 via standard restriction/ligation techniques. All constructs were confirmed by DNA sequencing. Bacterial expression vectors used for the *in vitro* interaction assays were: pDEST-GFP-RfB-β-DG-NLS encoding GFP fused to the NLS of β-DG [12], vectors encoding GST (glutathione S-transferase) alone or fused to mouse IMPs (described in [18]-[20]) and a pGTH-ezrin vector encoding GST fused to ezrin. This vector was engineered using the GFP-ezrin vector as template and the following oligonucleotides: forward 5′ GGCCAAGAATTCCCGAATGCCGAAACCAATC 3′ and reverse 5′ CCGGTTGTCGACACAGGGCCTCGAACTCG 3′, containing *SalI* and *EcoRI* restriction sites respectively. The PCR product was digested with *SalI* and *EcoRI,* cloned into the pGTH vector and confirmed by sequencing. psi-mH1 vector expressing an interfering RNA (RNAi) specific for IMPβ1 (Cat. # MSH027440-mH1), or an irrelevant RNAi (Cat. # CSHCTR001-mH1) and co-expressing the reporter protein mCherry for identification of transfected cells were purchased from GeneCopoeia, Rockville, MD USA.

### Immunofluorescence and confocal microscopy analysis

Cells were fixed with 4% paraformaldehyde for 10 min and permeabilized with 0.2% Triton X-100/PBS for 10 min. Cells were incubated overnight at 4°C with primary anti-β-DG antibody (C-20). The following day, cells were washed with PBS and incubated for 1 h at room temperature with secondary Alexa Fluor 594 chicken anti-goat IgG (1∶50, Invitrogen). For double labeled samples, this was followed by incubation overnight at 4°C with primary anti-ezrin antibody (H276). The next day, cells were incubated with secondary FITC-conjugated goat anti-rabbit IgG (1∶25; Zymed Laboratories, Inc., San Francisco, CA, USA) for 1 h at room temperature and counterstained with DAPI (0.2 µg/µl, Sigma-Aldrich) for 20 min at room temperature to label the cell nuclei. Where indicated F-actin was labelled using TRITC conjugated Phalloidin (Sigma-Aldrich St. Louis, Mo. USA) diluted 1∶500 in PBS for 10 min at room temperature. Finally, cells were washed with PBS, mounted on microscope slides with VectaShield (Vector Laboratories, Inc., Burlingame, CA, USA) and examined on a confocal laser scanning microscope (TCP-SP5, Leica, Heidelberg, Germany), employing a Plan Neo Fluor 63x (NA  =  1.4) oil-immersion objective. Image analysis of digitized confocal microscopic files was performed as previously [12], using the ImageJ 1.62 software to determine the nuclear to cytoplasmic fluorescence ratio (Fn/c) according to the formula Fn/c  =  (Fn-Fb)/(Fc-Fb), where Fn  =  nuclear fluorescence, Fc  =  cytoplasmic fluorescence and Fb  =  background fluorescence. Statistical significance was determined using a student’s- t-test.

### Immunoprecipitation assays

Recombinant protein G-agarose beads (8 µl per sample) (Invitrogen) were equilibrated in 500 µl of lysis buffer (50 mM Tris-HCl pH 8.0, 150 mM NaCl, 1 mM PMSF, 1% v/v Triton X-100) and shaken gently for 4 h at 4°C. Total extracts (1 mg) were pre-cleared with equilibrated beads for 2 h at 4°C, the beads were removed by centrifugation at 10,000 rpm for 5 min and the pre-cleared extracts incubated overnight at 4°C with the appropriate antibody. Parallel incubations with an irrelevant IgG antibody were also performed. 8 µl of equilibrated protein G-agarose beads blocked with 4% BSA were added to each sample and incubated overnight at 4°C. The immune complexes were collected by centrifugation at 10,000 rpm for 5 min, washed three times 10 min with 500 µl of wash buffer (50 mM Tris-HCl pH 8.0, 500 mM NaCl, 1 mM EDTA pH 8.0, 1% (v/v) Triton X-100, and 0.5 mM PMSF) and eluted by boiling in 10 µl of milli-Q H2O and 10 µl of SDS sample buffer (50 mM Tris-HCl pH 6.8, 2% (w/v) SDS, 10% (v/v) glycerol, 0.1% (v/v) 2-mercaptoethanol, 0.001% (w/v) bromophenol blue). Immunoprecipitated proteins were analyzed by western blotting.

### Bacterial protein expression and purification

GFP-β-DGNLS was purified from bacteria as His_6_-tagged proteins using nickel affinity chromatography under native conditions. Briefly, BL21 (DE3) bacteria were transformed to express the fusion protein, grown to OD_600_  =  0.6 and induced overnight with 1 mM IPTG. The bacteria was centrifuged at 14,000 rpm and the pellets resuspended in His-lysis buffer (8 M Urea, 0.1 M NaH_2_PO_4_, 0.01 M Tris and 1 M NaCl) containing 3 mg/ml lysozyme and lysed on ice for 30 min in the presence of 1 unit/ml DNase1 and Complete EDTA-free protease inhibitors (Roche Applied Science). The lysate was centrifuged at 11,000 x g for 45 min at 4°C and the soluble fraction incubated with 4 ml of pre-equilibrated Ni-NTA bead slurry (Qiagen) for 1 h at 4°C. The beads were washed and the protein was eluted in His-Lysis buffer containing 200 mM imidazole, followed by dialysis against PBS. The protein was further purified by gel filtration using a HiPrep 26/60 Sephacryl S-200 high resolution column attached to an ÄKTA Purifier system (GE Healthcare) and concentrated using Amicon centrifugal concentration devices (Millipore Corporation, Billerica, MA, USA). Protein concentration was estimated by absorbance measurement at 280 nm and the theoretical molar extinction coefficient.

GST-ezrin (mouse), GST-IMPα, GST-IMPβ and GST alone were purified from bacteria under native conditions, as described previously [21].

### ALPHAScreen assay

Interaction between GFP-β-DGNLS and ezrin or IMPα2/β1 heterodimer was assessed using an established ALPHAScreen assay (Perkin Elmer, Wellesley,MA) [21], whereby IMPs α2 and β1 were pre-dimerized at 13.6 µM for 15 min at room temperature in intracellular buffer (20 mM HEPES pH 7.4, 110 mM KCl, 5 mM NaHCO_3_, 5 mM MgCl_2_, 1 mM EGTA, 0.1 mM CaCl_2_, 1 mM DTT). Briefly, 30 mM of His_6_-tagged GFP-β-DG-NLS fusion protein per well in a 384-well white opaque plate (Perkin Elmer) were incubated for 30 min with increasing concentrations of GST-ezrin or GST-IMPs (0–60 nM). 1 µl of a 1∶10 dilution of Nickel chelate acceptor beads and 1 µl of 2.5% BSA were added and incubated at room temperature for 90 min, followed by addition of 1 µl of a 1∶10 dilution of the streptavidin donor beads and incubation at room temperature for 2 h. Analysis was carried out using a FusionAlpha plate reader (Perkin Elmer).

### Cell fractionation

To isolate cytosolic and nuclear extracts, cells were washed twice with 2 ml of ice-cold PBS and collected by centrifugation at 6,000 rpm for 15 min at 4°C. The pellet was resuspended in TM buffer (10 mM Tris-HCl pH 8.0, 2 mM MgCl_2_, 0.5 mM PMSF) supplemented with 1X complete protease and phosphatase inhibitors (2 mM Na_3_VO_4_, 10 mM Na_2_MoO_4_, and 25 mM NaF) and incubated for 10 min on ice. 2% Triton X-100/PBS was added and the homogenate incubated for 10 min on ice. Nuclei were separated from the cytosol using a glass Dounce homogenizer (30 strokes using the B pestle) and centrifuged at 6,000 rpm for 15 min at 4°C. The supernatant contains the cytosolic fraction and the nuclear pellet was resuspended in 1 ml of buffer I (0.32 M Sucrose, 3 mM CaCl_2_, 2 mM Mg(COO)_2_, 0.1 mM EDTA, 10 mM Tris-HCl pH 8.0, 1 mM DTT, 0.5 mM PMSF, 0.5% (v/v) NP40) and 1 ml of buffer II (2 M Sucrose, 2 mM Mg(COO)_2_, 0.1 mM EDTA, 10 mM Tris-HCl pH 8.0, 1 mM DTT, 0.5 mM PMSF) and further purified by centrifugation at 16,000 rpm through a sucrose gradient for 1 h at 4°C. Nuclei were recovered using lysis buffer (50 mM Tris-HCl pH 8.0, 150 mM NaCl, 1 mM PMSF, 1% (v/v) Triton X-100), supplemented with protease inhibitor cocktail and phosphatase inhibitors, vortexed for 30 min at 4°C, sonicated at 4 microns for 2 min and pre-cleared at 13,000 rpm for 2 min at 4°C.

### Western blotting

Protein samples (80 µg) were electrophoresed on 10% SDS-polyacrylamide gels and transferred to nitrocellulose membranes (Hybond-N+, Amersham Pharmacia, GE Healthcare, Buckinghamshire, UK) using a Transblot apparatus (Bio-Rad, Hercules, CA, USA). Membranes were blocked for 1 h in TBS-T (10 mM Tris-HCl pH 8.0, 150 mM NaCl, 0.05% (v/v) Tween-20) with 6–15% (w/v) skim milk. Membranes were incubated overnight at 4°C with the appropriate primary antibody. After three washes in TBS-T, membranes were incubated with the corresponding horseradish peroxidase-conjugated secondary antibody (Amersham Pharmacia, GE Healthcare, Buckinghamshire, UK) and developed using the ECL Western blotting analysis system (Amersham Pharmacia, GE Healthcare, Buckinghamshire, UK).

### Biotinylation of cell surface proteins

Cells were transfected with the appropriate plasmids and 24 h post-transfection surface proteins were biotinylated with 0.25 mM sulfo-NHS-LC-biotin (Thermo-Fisher Scientific, Rockford, IL) at room temperature for 30 min, followed by 100 mM glycine in PBS to stop the reaction, as described previously [22]. Treated cells were processed for cell fractionation and the nuclear and cytoplasmic extracts (250 µg) precipitated overnight at 4°C using 20 µl of Streptavidin-agarose beads (Thermo-Fisher Scientific, Rockford, IL). Beads were collected by centrifugation at 10,000 rpm for 5 min at 4°C, washed tree times with ice cold 0.1% Triton X-100 in PBS and the precipitates solubilized in 6X SDS-PAGE sample loading buffer and boiled for 10 min prior to Western blotting.

## Results

### Ezrin colocalizes and interacts with β-DG in C2C12 myoblasts

To ascertain whether β-DG co-localizes and associates with ezrin, C2C12 myoblasts were analyzed by confocal microscopy and immunoprecipitation. Ezrin and β-DG exhibited similar localization patterns as each other, with both proteins found to localize in different cellular compartments, including the plasma membrane, cytoplasm and to a lesser extent the nucleus (Fig. 1B). Colocalization of β-DG with ezrin was apparent, especially at the cell surface projections (inset), suggesting that the two interact in this cell system. Immunoprecipitation analysis showed that ezrin co-immunoprecipitated with β-DG using anti-β-DG antibodies, confirming that β-DG interacts with ezrin in these cells (Fig. 1C). Neither of the proteins were immunoprecipitated when an irrelevant antibody (IgG0) was used as a control, indicating the specificity of this assay. Since the IMPα2/β1-recognized nuclear localization signal (NLS) of β-DG [12] also serves as the ezrin-binding site [2], it is plausible to hypothesize that ezrin may compete with IMPα2/β1 for binding to the β-DG NLS, and thereby interfere with its nuclear import. To begin to examine this we compared the binding affinities of the IMPα2/β1 heterodimer and ezrin for the NLS of β-DG using an ALPHAScreen protein-protein interaction assay (see Material and Methods). The IMPα2/β1 heterodimer bound with higher affinity (Fig. 1D, Kd  =  0.98 nM) than ezrin (Kd  =  2.22 nM) to the NLS of GFP-β-DGNLS. As expected no binding was observed using GST alone (negative control).

### Overexpression of active ezrin induces reorganization of the actin cytoskeleton and nuclear accumulation of β-DG

**Figure 2:**
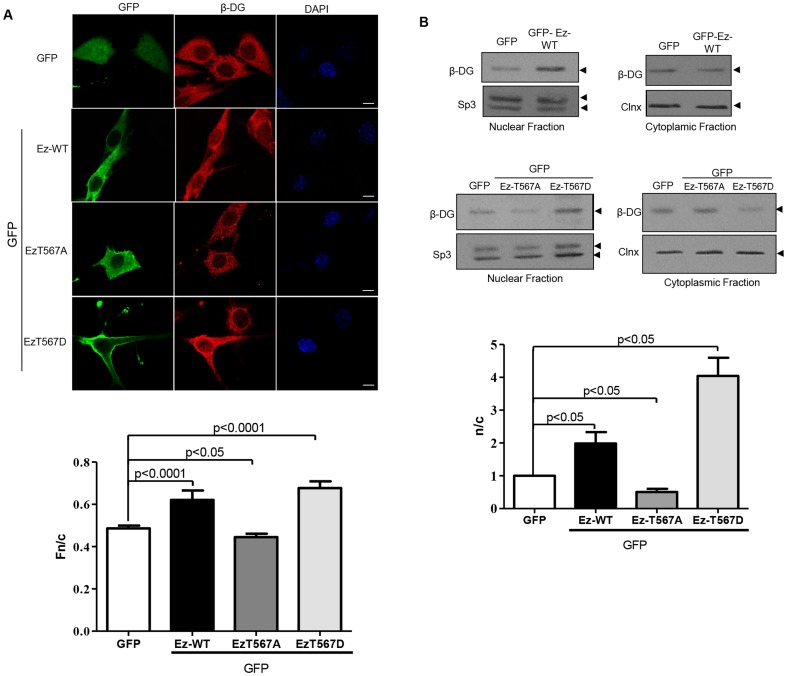
Overexpression of active ezrin facilitates nuclear translocation of β-DG. **A.** C2C12 myoblasts cultured on glass coverslips were transfected to express ezrin-GFP (Ez) fusion proteins (either wild type, WT, or the mutated variants T567D and Ez-T567A) or GFP alone. Cells were fixed and stained 24 h post-transfection with a polyclonal anti-β-DG antibody (JAF) and TRITC-conjugated secondary antibody, with nuclei stained using DAPI (blue). Cells were imaged by CLSM, with typical single Z-sections shown (scale bar is 10 µm). Quantitative analysis for the nuclear to cytoplasmic ratio (Fn/c) of β-DG was performed (bottom panel) using the Image J software, as described in Material and Methods. Results represent the mean +/– SD (n > 50 cells) from a series of three separate experiments, with significant differences between cells expressing GFP alone and cells expressing the different GFP-tagged ezrin variants determined by Student t-test. **B.** Cytoplasmic and nuclear extracts obtained from cells transfected to express the above constructs were separated by SDS-PAGE and subjected to Western analysis for β-DG. Membranes were stripped and reprobed for Sp3 and calnexin (Clnx); loading controls for nuclear and cytoplasmic extracts respectively. Densitometric analysis of autoradiograms was performed, and the nuclear/cytoplasmic ratio (n/c) for β-DG obtained by dividing the relative levels of β-DG in the nuclear extracts with those obtained in the corresponding cytoplasm extracts (bottom panel). Results represent the mean +/– SD for 3 separate experiments, with significant differences between cells expressing GFP alone and those expressing the different GFP-tagged ezrin variants determined by Student t-test.

To examine whether ezrin acts as a negative regulator of β-DG nuclear accumulation C2C12 cells were transfected to express various GFP-tagged ezrin mutants, including wild type ezrin (Ez-WT) and two ezrin variants bearing mutations at threonine 567 (T567), which either mimic (Ez-T567D) or block (Ez-T567A) phosphorylation at this site, which would render the protein active and able to bind to F-actin [23]. Immunofluorescence analysis of transfected cells demonstrated that overexpression of Ez-Wt or Ez-T567D (active ezrin) but not GFP alone or Ez-T567A (inactive ezrin) resulted in the induction of actin-rich cell surface protrusions (Fig. S1, panel A). Interestingly, the cytoskeletal reorganization occurring in cells expressing Ez-WT or Ez-T567D was accompanied by increased nuclear localisation of β-DG, compared with cells expressing Ez-T567A or GFP alone (Fig. 2A; upper panels). Quantitative analysis confirmed these observations, where image analysis used to determine the nuclear to cytoplasmic fluorescence ratio (Fn/c), indicated significantly increased nuclear localisation of β-DG in cells transfected with either Ez-WT or Ez-T567D (Fn/c of 0.62 and 0.67 respectively), compared with those expressing Ez-T567A or GFP alone (Fn/c of 0.44 and 0.48 respectively) (Fig. 2A; bottom panel). To examine this further, C2C12 cells transfected with the indicated ezrin constructs were fractionated into cytosolic and nuclear extracts and analyzed by immunoblotting using an anti-β-DG antibody (Fig. 2B). The purity of the subcellular fractions was routinely monitored by using antibodies against Sp3 and/or lamin B1 (nuclear markers) or GAPDH and/or calnexin (cytoplasmic markers) (see Fig. S2), and only once the purity of the extracts was established, did we proceed with immunodetection of β-DG using a new membrane, which was used for immunodetection of the corresponding loading control (calnexin for cytoplasmic extracts and Sp3 for nuclear extracts) after stripping of the membrane. Consistent with the IF assays, a significant increase in the nuclear level of β-DG was observed in cells exogenously expressing Ez-WT, compared with cells expressing GFP alone (Fig. 2B; upper panels). Importantly, nuclear accumulation of β-DG appeared to be even greater in cells expressing Ez-T567D, while in contrast, overexpression of Ez-T576A resulted in decreased levels of β-DG in the nucleus, compared with GFP alone (Fig. 2B; middle panels). Accordingly, quantitative analysis revealed that the nuclear to cytoplasmic ratio (n/c) of β-DG increased ∼2 and 4-fold in cells expressing Ez-WT and Ez-567D respectively, while a modest reduction was observed with expression of Ez-T576A (Fig. 2B; bottom panel). Overall these results demonstrate that exogenously expressed ezrin in its activated state facilitates the nuclear accumulation of β-DG.

### Rho-mediated ezrin activation facilities nuclear translocation of β-DG

**Figure 3:**
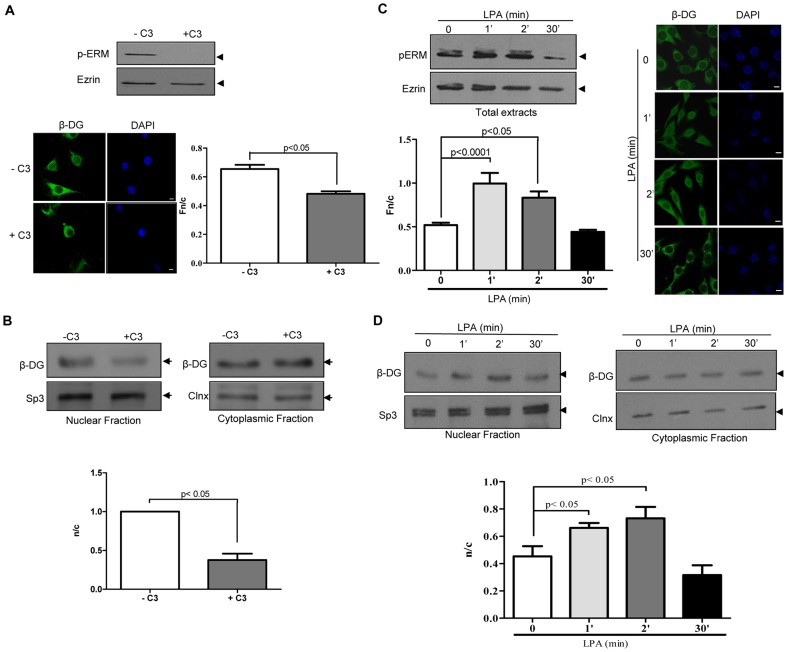
Rho signaling-mediated activation of ezrin facilitates nuclear accumulation of β-DG. **A.** Treatment of C2C12 cells with the toxin C3, a specific Rho inhibitor, decreased β-DG nuclear accumulation. Lysates from cells treated or not with C3 were analyzed by SDS/Western blotting using an antibody that recognizes phosphorylated ezrin, raxidin and moesin (p-ERM). The membranes were reprobed with antibody for total ezrin (upper panel). Cells grown on coverslips were treated without or with C3, fixed and immunolabeled using anti-β-DG primary and a fluorescein-conjugated secondary antibodies and nuclei stained with DAPI. Cells were imaged and quantitative analysis for Fn/c of β-DG was performed as described in Figure 2 (bottom right panel) showing significant differences between cells treated or not with C3. **B.** Nuclear and cytoplasmic fractions from control and C3-treated cells were analyzed by SDS/Western blotting using anti- β-DG antibodies (upper panels). Immunodetection of Sp3 and Calnexin (Clnx) was used as loading control for nuclear and cytoplasmic fractions respectively. The n/c ratio for β-DG was quantified and plotted as per Figure 2 (bottom panel) with significant differences determined by *p* value. **C.** C2C12 cells treated with LPA (inductor of the Rho pathway) show increased β-DG nuclear accumulation. Lysates from cells pretreated with LPA or PBS for 0–30 min were analyzed by SDS/Western blotting using anti-p-ERM or anti-ezrin antibodies (upper left panel). Control and LPA-treated cells were immunolabeled for β-DG and imaged as before (left panel) with significant differences between control and treated cells. **D.** Cytoplasmic and nuclear extracts of untreated and LPA-treated cells were analyzed by SDS/Western blotting using anti-β-DG antibodies. Sp3 and Calnexin were employed as loading controls (upper panels). The n/c ratio for β-DG shows significant differences between control and treated cells. All results representing the mean +/- SD from three separate experiments were analyzed by Student t-test.

If activation of ezrin plays a role in the nuclear translocation of β-DG, it should be expected that alteration of the signaling cascade that results in phosphorylation of ezrin on T567, the Rho signaling pathway, will also alter the nuclear accumulation of β-DG. To test this idea, C2C12 cells were treated with the bacterial toxin C3, a specific Rho inhibitor, prior to analysis of the subcelular distribution of β-DG as previously. Lysates from control and C3-treated cells were analyzed by Western blot analysis using either a pan-antibody directed against phosphorylated ezrin/radixin/moesin (pERM) or with antibody that recognizes ezrin irrespective of its phosphorylation state. Treatment with C3 ablated phosphorylation of ERM, while maintaining unaltered total levels of ezrin (Fig. 3A, upper panel), demonstrating the effectiveness of the treatment. Consistent with our hypothesis, confocal microscopy analysis of C3-treated cells showed disruption of the actin cytoskeleton (Fig. S1, panel B) and a concomitant decrease in the localisation of β-DG in the nucleus, compared with untreated cells (Fig. 3A, bottom left panel). Quantitative analysis confirmed these observations, indicating significantly decreased nuclear accumulation of β-DG in C3-treated cells, compared with control cells (Fn/c of 0.65 and 0.48 respectively; Fig. 3A, bottom right panel). Consistent with this, cell fractionation of C2C12 cells into cytosolic and nuclear extracts revealed a significant reduction in the nuclear levels of β-DG upon C3 treatment (Fig. 3B, upper panels), with quantitative analysis indicating a n/c ratio of 1.00 and 0.37 for control and treated cells respectively (Fig. 3B, bottom panel). Similarly, activation of the Rho pathway through stimulation of C2C12 cells with lysophosphatidic acid (LPA) transiently augmented the levels of phosphorylated ERM at 1 and 2 min with a decline observed by 30 min (Fig. 3C, upper panel). Rho pathway activation also resulted in the formation of actin-rich cell surface protrusions (Fig. S1, panel C), as well as in a transitory increase in the nuclear localization of β-DG that followed the same time course as ERM activation; increased nuclear accumulation of β-DG in cells treated with LPA for 1 and 2 min that had reverted by 30 min of treatment, compared with untreated cells (Fig 3C, right), with quantitative analysis consistent with these observations (Fig. 3C, bottom left). In concordance, immunoblotting analysis of cytosolic and nuclear extracts of C2C12 cells showed an increase in the nuclear levels of β-DG in cells treated with LPA for 1 and 2 min that disappeared after 30 min of treatment (Fig. 3D, upper panels), with quantitative analysis indicating significant differences in the n/c ratio of β-DG between control cells and cells treated with LPA for 1 and 2 min, but not 30 min (Fig. 3D, bottom).

### Ezrin-dependent nuclear translocation of β-DG is mediated by cytoskeletal reorganization

**Figure 4:**
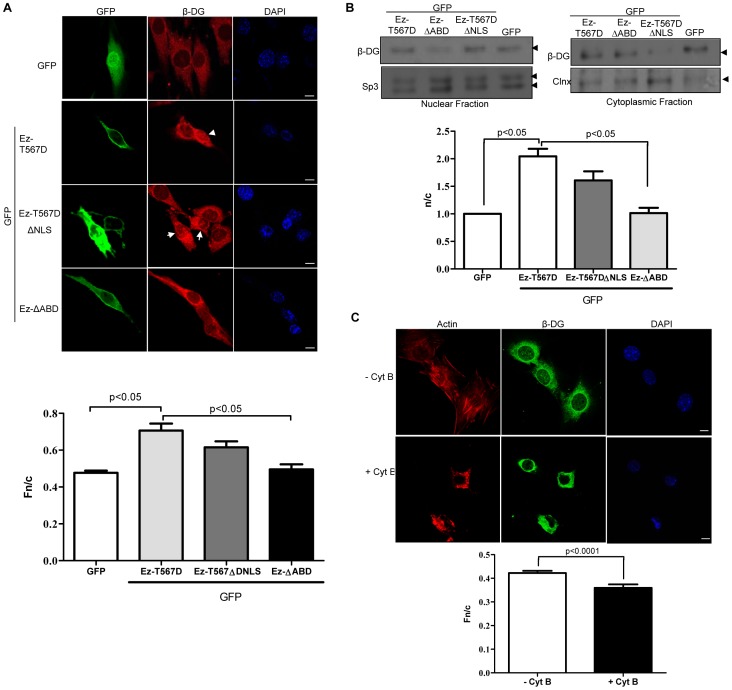
Ezrin-mediated cytoskeleton reorganization facilitates nuclear translocation of β-DG. **A.** C2C12 myoblasts grown on coverslips were transfected to express GFP-tagged-EzT567D (active ezrin), -EzT567ΔNLS (active ezrin carrying a deletion of the NLS), or -EzΔABD (ezrin variant with a deletion of the actin-binding domain), or GFP alone. Transfected cells were immunostained with anti-β-DG primary and TRITC-conjugated secondary antibodies and counterstained with DAPI (nuclei), prior to CLSM, with typical single Z-sections shown (scale bar is 10 µm). Images were analysed to determine the Fn/c for β-DG (bottom), as per Figure 2. Results represent the mean +/– SD (n > 50) from three separate experiments, with significant differences in the Fn/c of β-DG between cells expressing GFP alone and those expressing GFP-Ez-T567D, and between cells expressing GFP-Ez-T567D- and GFP-EzΔABD, as denoted by the p values. **B.** Cytoplasmic and nuclear extracts from transfected cells with the above constructs were analyzed by SDS/Western blotting using anti-β-DG antibodies. Sp3 and calnexin (Clnx) were immunodetected as loading controls for nuclear and cytoplasmic fractions respectively. Densitometric analysis was performed to obtain the n/c ratio of β-DG for the different transfected cultures (right panel), as per Figure 2. Results represent the mean +/- SD for 3 separate experiments, with significant differences between cells expressing GFP alone and those expressing GFP-Ez-T567D, as well as between cell expressing GFP-Ez-T567D and those expressing GFP-EzΔABD, as denoted by the p values. **C.** C2C12 myoblasts seeded in coverslips were treated without (control) or with 6 µM cytochalasin B (Cyt B) for 1 h. Treated cells were fixed and double-stained with anti-β-DG primary antibody and fluorescein-conjugated secondary antibody, and with TRITC-phalloidin to visualize effects on the actin-based cytoskeleton network. Nuclei were counterstained with DAPI. Samples were imaged and subjected to image analysis as described in Figure 2. Results represent the mean +/- SD for three separate experiments (n> 50), with p values determined by Student t-test denoting significant differences between control and Cyt B-treated cells (bottom panel).

Since ezrin is imported into the nucleus via a nuclear localization signal (NLS) located in its α-domain, we speculated that increased nuclear translocation of β-DG mediated by active ezrin may occur through a piggy-back mechanism utilising the NLS of ezrin. Although β-DG can be imported into the nucleus in an importin α2/β1-dependent fashion through its own NLS [12], an alternative nuclear import pathway may serve to improve its nuclear accumulation. To evaluate this hypothesis, C2C12 myoblasts were transfected to express either GFP alone, active ezrin (Ez-T567D) or a mutant variant lacking the NLS (Ez-T567DΔNLS) and distribution of β-DG examined by confocal microscopy and western blot analysis of cytoplasmic and nuclear extracts. Increased nuclear accumulation of β-DG was observed in cells expressing GFP-EZ-T567D, compared with GFP alone (Fig. 4A) with Fn/c values of 0.71 and 0.47 respectively (bottom panel), as showed above (Fig. 2A). Cells expressing GFP-Ez-T567D or GFP-Ez-T567DΔNLS revealed similar intensity in the nuclear labeling of β-DG (Fig. 4A), with quantitative analysis of confocal microscopy images confirming these observations (Fn/c values for GFP-Ez-T567D and -T567DΔNLS of 0.71 and 0.62 respectively; bottom panel). Results for the fractionation of cells into cytoplasmic and nuclear extracts and Western analysis for β-DG were consistent with these results; nuclear levels of β-DG were increased to a similar extent in response to overexpression of GFP-Ez-T567D or -T567DΔNLS, compared with GFP alone (Fig. 4B), with n/c values for GFP-Ez-T567D and -T567DΔNLS of 2.0 and 1.6 respectively (Fig. 4B bottom panel), compared to a value of 1 for GFP alone. These results indicate that active ezrin-mediated nuclear translocation of β-DG is not dependent on nuclear import of ezrin.

Although critically dependent on interactions with IMPs, nuclear protein import has also been shown in many cases to be strongly influenced by interactions with cytoskeletal elements [15], [24]. Since ezrin is involved in the organization of the cortical cytoskeleton, we hypothesized that nuclear accumulation of β-DG in response to active ezrin might be an effect of the ezrin-mediated cytoskeleton reorganization on the nuclear import pathway of β-DG. Hence, we investigated whether overexpression of an ezrin mutant variant lacking the actin-binding domain (Ez-ΔABD; 1-470 aa) influences the nuclear translocation of β-DG. C2C12 myoblasts expressing Ez-ΔABD exhibited decreased nuclear accumulation of β-DG, compared with cells expressing Ez-T567D (Fig. 4A; Fn/c of 0.49 and 0.71 respectively). Consistently, western blot analysis of cytoplasmic and nuclear extracts revealed reduced levels of β-DG in the nucleus of Ez-ΔABD-transfected cells, compared with those expressing Ez-T567D (Fig. 4B, left panels). Densitometric analysis corroborated these observations, with n/c values for Ez-T567D being significantly greater than that of Ez-ΔABD (2.1 and 1.01 respectively; Fig. 4B, right panel). These results demonstrate that ezrin requires its actin-binding domain to facilitate nuclear translocation of β-DG, implying that nuclear import of β-DG is modulated by the actin-based cytoskeleton. To test this prediction, C2C12 myoblasts were treated with the actin-depolymerizing agent cytochalasin B, fixed, stained with phalloidin and counterstained with anti- β-DG antibodies. Cytochalasin B-induced disorganization of the actin network (Fig. 4C), resulted in a significant decrease in the nuclear localisation of β-DG, compared with untreated cells. These results are consistent with the idea that nuclear translocation of β-DG is dependent on actin-based cytoskeletal integrity.

### Ezrin-induced nuclear translocation of β-DG occurs through the importin nuclear import pathway

**Figure 5:**
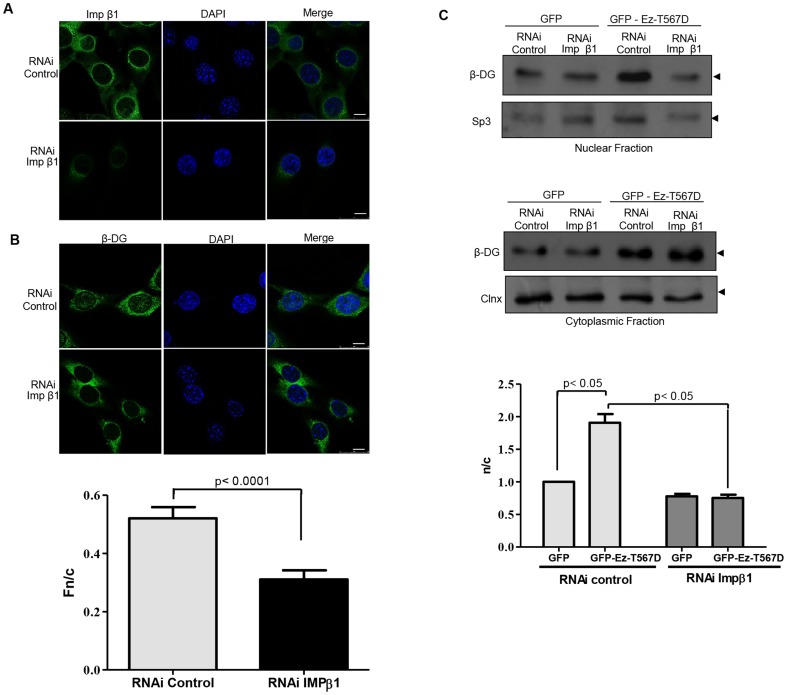
Nuclear translocation of endogenous β-DG induced by active ezrin is dependent on IMPβ1. C2C12 myoblasts stably transfected with vector expressing either the control or importin β1 (IMPβ1) RNAi were cultured on glass coverslips, fixed and immunostained for IMPβ1 **A** or β-DG **B**, using FITC-conjugated secondary antibody (green), with nuclei stained using DAPI (blue). Cells were imaged by CLSM, with typical single Z-sections shown (scale bar is 10 µm). **B.** Quantitative analysis to determine the nuclear to cytoplasmic ratio (Fn/c) of β-DG was performed in control- and RNAi IMPβ1-transfected cells (bottom panel), as per the legend to Figure 2. Results represent the mean +/– SD (n > 50 cells) from a series of three separate experiments, with significant differences between cells expressing the control or IMPβ1 RNAi determined by Student t-test. **C.** Cytoplasmic and nuclear fractions obtained from cells stably expressing either the control or IMPβ1 RNAi and transiently expressing GFP or Ez-T567D-GFP fusion proteins were analyzed by SDS-PAGE/Western using an anti-β-DG antibody (upper panels). Membranes were stripped and reprobed with antibodies against calnexin (Clnx) and Sp3, loading controls for cytoplasmic and nuclear lysates respectively. Nuclear to cytoplasmic ratio (n/c) of β-DG levels were quantified and plotted (bottom panel), as per the legend to Figure 2. Results represent the mean +/– SD from a series of three separate experiments, with significant differences between cells expressing the control or IMPβ1 RNAi determined by Student t-test.

We previously demonstrated that nuclear import of β-DG is mediated by IMPα2/β1 through recognition of its NLS [12]. To determine whether ezrin-mediated nuclear accumulation of β-DG is also dependent on the IMP nuclear import pathway, C2C12 cells were stably transfected with a vector expressing a small interfering RNA (RNAi) targeting IMPβ1, using an RNAi that is predicted not to block the translation of any specific gene as a negative control (control RNAi). Effectiveness of the RNAi treatment in reducing IMPβ1 was evaluated by indirect immunofluorescence. Global immunolabeling of IMPβ1 was drastically reduced in cells expressing IMPβ1-specific RNAi, compared with cells expressing the control RNAi (Fig. 5A). IMPβ1-depeleted cells were transiently transfected with vectors expressing GFP (control) or active ezrin (Ez-T567D) and analysed by confocal microscopy 24 h post-transfection. Ablation of IMPβ1 expression resulted in a drastic reduction of β-DG nuclear staining, compared with control RNAi cells (Fig. 5B, Fn/c of 0.31 and 0.52 respectively). Consistent with the idea that nuclear translocation of β-DG induced by ezrin activation is dependent on IMPβ1, SDS/Western blot analysis of cytoplasmic and nuclear extracts revealed that the increased nuclear levels of β-DG that resulted from Ez-T567D overexpression were not observed in IMPβ1-depleted cells (Fig. 5C), with quantitative analysis showing significant differences in the n/c ratio of β-DG between RNAi control and RNAi IMPβ1-expressing cells (bottom panel).

### Ezrin-dependent cytoskeleton reorganization enhances nuclear translocation of the cytoplasmic but not the transmembranal fraction of β-DG

**Figure 6:**
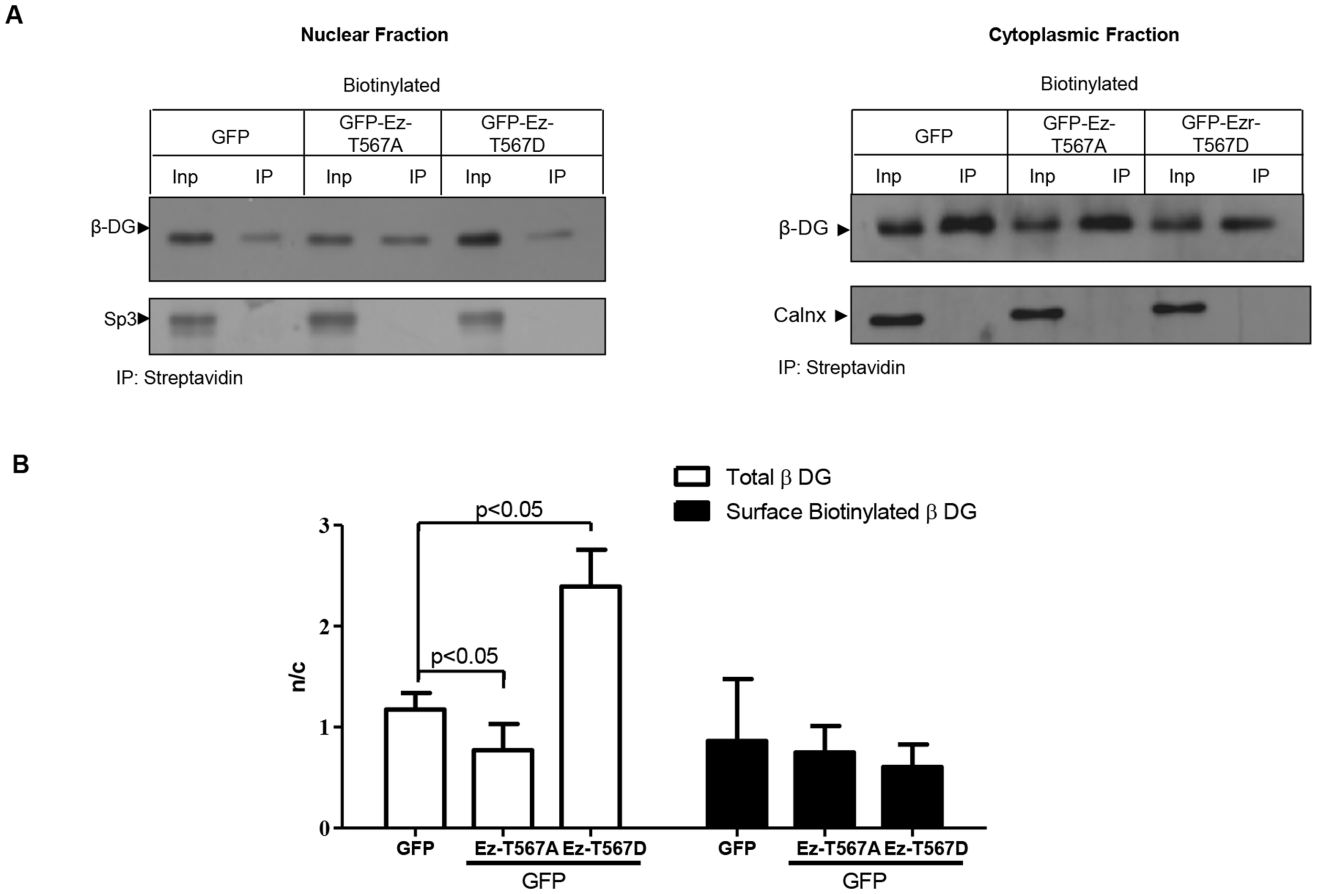
Subcellular distribution of cell surface-biotinylated β-dystroglycan in C2C12 myoblasts overexpressing active ezrin. **A.** Cells were transfected to transitory express either ET567D-GFP, Ez-T567A-GFP or GFP alone and incubated with biotin 24 h post-transfection to label cell surface proteins, as described in Material and Methods. Cytosolic and nuclear fractions isolated from biotinylated cells were pulled-down using streptavidin-agarose beads and precipitated proteins were subjected to SDS-PAGE/Western analysis using an anti-β-DG antibody (7D11). Input, biotinylated cytosolic and nuclear extracts subjected to SDS-PAGE/Western analysis without previous streptavidin-mediated precipitation. Membranes were stripped and reprobed for calnexin (Clnx) and Sp3, loading controls for cytoplasmic and nuclear lysates respectively. **B.** Nuclear to cytoplasmic levels (n/c) of β-DG were quantified as per the legend to Figure 2 and results plotted represent the mean +/- SD from a series of three separate experiments, with significant differences determined by Student t-test.

We recently found that at least a fraction of nuclear β-DG comes from the plasma membrane (unpublished data). Therefore, we were intrigued to know if the plasma membrane is the origin of the β-DG that is translocated to the nucleus in response to ezrin activation. To approach this, C2C12 cells transiently transfected to express either GFP (control), active (ET567D) or inactive (E-T567A) ezrin were incubated with biotin for 30 min to label cell surface proteins and then fractionated into cytosolic and nuclear extracts. Biotinylated proteins were precipitated with streptavidin-agarose beads, resolved by SDS/PAGE and analyzed by immunoblotting with anti-β-DG antibodies. The presence of biotinylated β-DG in the nucleus identifies the fraction of β-DG translocated from the plasma membrane to the nucleus. Protein extracts that were not subjected to streptavidin-mediated pull-down were analyzed simultaneously to estimate the nuclear levels of β-DG irrespective of its subcellular origin. When the protein extracts were not precipitated with streptavidin-agarose beads, overexpression of active ezrin resulted in an augmentation of the levels of nuclear β-DG, compared with GFP and inactive ezrin, as previously (Fig. 6A; Input lanes), with quantitative analysis confirming this result (Fig. 6B). Interestingly, when biotinylated β-DG was specifically analyzed, no increase in the nuclear levels of β-DG was found in response to overexpression of active ezrin (Ez-T567D), compared with inactive ezrin (Ez-T567A) or GFP alone (Fig. 6A), with quantitative analysis (n/c) supporting these observations (Fig. 6B). Overall these results suggest that the fraction of β-DG imported to the nucleus upon ezrin activation did not come from the plasma membrane but rather from a cytoplasmic pool.

## Discussion

Although β-DG is a transmembrane component of the dystrophin associated protein complex involved in cell adhesion and cytoskeleton remodeling [2], [5], it has the capability to translocate to the nucleus via the canonical IMPα2/β1 nuclear import pathway, using a functional NLS located in the juxtamembrane region (776–782 aa) [11], [12]. As a nuclear protein β-DG is localized to the nuclear envelope, where it is involved in nuclear architecture and nuclear envelope-associated functions through binding to lamins A/C and B1 and emerin [13]. Therefore, β-DG is currently regarded as multi-functional protein playing a variety of functions in different cellular compartments, which implies that a balance of β-DG levels between the plasma membrane and the nucleus must be achieved to properly orchestrate its adaptive response to the functional requirements of the cell. However, besides the critical participation of IMPα2/β1, the molecular mechanisms modulating nuclear trafficking of β-DG are largely unknown. In particular, the accessibility of the NLS to IMPα2/β1 might be a mechanism modulating β-DG nuclear import as the cluster of basic amino acids constituting the NLS also serves as the ezrin binding site [2]. Ezrin is involved in a variety of cellular functions, including cell adhesion, migration, and organization of cell surface structures [25], [26] and works in concert with β-DG to induce peripheral filopodia and microvilli [2], [3]. One important mechanism regulating the functioning of ezrin is phosphorylation at a conserved threonine residue in the C terminus (Thr^567^). Ezrin usually exists in a folded conformation due to head-to-tail interactions between amino and carboxyl-terminal ERM association domains, masking its binding sites from other molecules. However, phosphorylation of this conserved threonine residue by PKC-θ or Rho kinase results in the relief of this autoinhibitory interaction [23], [27], holding ezrin in an open conformation and thus in an active state.

In this study we used C2C12 myoblasts as a model for testing the hypothesis that ezrin could regulate the nuclear translocation of β-DG, assuming the premise that binding of ezrin to the NLS of β-DG might block access of IMPα2/β1 to this motif, restraining its nuclear import. We show here that β-DG displays a rather disparate diverse subcellular distribution in C2C12 cells, being able to localize at the plasma membrane, as well as in the cytoplasm and nucleoplasm; similar results have been reported previously reported for other cell lines as well as primary cultures [2], [4], [5], [8]–[13], [28], [29]. This wide distribution can be explained in terms of the ability of β-DG to traffic within the cell, whereby subsequent to translation in the ER and post-translational modification in the Golgi to reach the plasma membrane, β-DG can be internalized into recycling endosomes [30]. This seems likely to enable subsequent trafficking events to the nucleus, as shown here; within the nucleus, β-DG localizes and interacts with distinctive proteins of the nucleoplasmic bodies nucleoli and Cajal bodies [13]. We demonstrate here that ezrin colocalizes with β-DG, with the interaction between these two proteins confirmed by IP. Interestingly, the IMPα2/β1 heterodimer interacts *in vitro* with the NLS of β-DG with higher affinity than ezrin. Unexpectedly, transfection of ezrin variants and experiments modulating activation of endogenous ezrin provided no evidence supporting the notion that ezrin restrains the nuclear import of β-DG. On the contrary, our data revealed that ezrin acts to enhance β-DG nuclear import through its induction of actin-based cytoskeletal reorganization. We found that exogenous expression of an ezrin mutant variant that mimics its activated phosphorylated state (Ez-T567D), as well as activation of endogenous ezrin through LPA-mediated stimulation of the Rho signaling pathway induced actin-rich surface protrusions in C2C12 myoblasts and caused a concomitant increase in the nuclear localization of β-DG, observed by both immunofluorescence and western blotting analyses of cytosolic and nuclear extracts. Consistently, reciprocal experiments where an ezrin mutant that is unable to be phosphorylated on threonine (Ez-567A) was overexpressed in C2C12 cells or where these cells were treated with the Rho pathway inhibitor C3 to prevent ezrin activation, caused a decrease in the nuclear accumulation of β-DG, evidenced by immunofluorescence and biochemical analysis of cell fractions, with no induction of cell surface structures. Compatible with the idea that ezrin enhances nuclear import of β-DG via reorganization of the actin-based cytoskeleton, overexpression of an ezrin variant that lacks the actin-binding domain failed to favor nuclear accumulation of β-DG, with disruption of the cytoskeleton by cytochalasin B treatment also causing a significant reduction of β-DG nuclear levels. Furthermore, we demonstrated that enhanced nuclear translocation of β-DG that occurs in response to ezrin activation is mediated by the β-DG NLS/IMP nuclear import pathway, as knock-down of IMPβ1 expression reduced nuclear translocation of β-DG, in spite of the overexpression of active ezrin (Ez-T567D).

One question arising from this study is how the ezrin-mediated cytoskeleton remodeling enhances nuclear translocation of β-DG in an IMP-dependent fashion. Recently, the actin cytoskeleton has been implicated in the movement of several proteins to the nucleus. For example, nuclear translocation of NF-κB occurs in response to rearrangements of the actin cytoskeleton and the formation of actin stress fibers in thrombin-activated endothelial cells [31]. Similarly, trafficking of WTIP (Wilms Tumor 1 Interacting Protein), a LIM (**L**in11, **I**sl-1 and **M**ec proteins) domain-containing protein, from adherent junctions to the nucleus is induced by lipopolysaccharide-mediated destabilization of the actin cytoskeleton in cultured podocytes [32]. Likewise, other members of the Ajuba LIM protein family, Ajuba, and LIM Domain-Containing Protein 1 (LIMD1), which link cell adhesive complexes to the cytoskeleton, have been shown to shuttle into the nucleus to regulate the activity of specific transcription factors [33]–[35]. It is thought that cytoskeletal transport acts as an enhancer of traditional nuclear import, accelerating protein transport through the cytoplasm to the nuclear periphery, where IMPs and conventional nuclear import mechanisms presumably take over [24]. With respect to β-DG, it has been shown that filopodia formation relies on the recruitment of ezrin and Db1 to the plasma membrane by β-DG [3], which implies that β-DG is inserted in the plasma membranes with its NLS bound to ezrin during this process. Therefore, it is intriguing how cytoskeleton reorganization mediated by ezrin could triggers nuclear translocation of β-DG in an NLS/IMP-dependent fashion. We propose that two different pools of β-DG are involved in this process; a transmembrane fraction of β-DG that is engaged together with ezrin in forming actin-rich filopodia, and therefore has its NLS inaccessible to IMPs, and a cytoplasmic pool of β-DG that is available for recognition by the IMP nuclear import machinery, to respond to ezrin activation. Supporting this hypothesis, we showed by biotinylation of cell surface proteins that trafficking of β-DG from the plasma membrane to the nucleus is not enhanced by ezrin-mediated cytoskeleton reorganization, indicating that it is indeed a separate cytoplasmic fraction of β-DG that accumulates in the nucleus upon ezrin activation.

In eukaryotic cells, signaling pathways in subcellular compartments must be integrated dynamically for a cell to respond appropriately to various environmental changes and functional requirements. The cytoskeleton often functions as a platform for signaling transduction in the cytoplasm and has been assumed to only indirectly contribute to nuclear signaling. However, there is increasing evidence that many types of plasma membrane and cytoskeleton proteins are also localized to the nucleus, suggestive of their direct involvement in the transmission of nuclear signaling and the regulation of nuclear functions (reviewed in [36]). In this regard, since β-DG is part of two main cellular complexes; the DAPC complex involved in functionally connecting the extracellular matrix and the cytoskeleton [2], [5] and a nuclear envelope complex implicated in nuclear architecture and function in myoblasts [13], it is tempting to propose that β-DG senses cytoskeleton-based changes in cell morphology and responds by translocating from the cytoplasm to the nucleus to orchestrate nuclear processes (i.e. nuclear architecture reorganization) in response to the new physiological conditions of the cell.

In summary, we show herein that trafficking of β-DG from the cytoplasm to the nucleus is enhanced by ezrin-mediated cytoskeleton reorganization in an IMPα2/β1-dependent fashion, which implies that β-DG might functionally link the cytoplasm with the nucleus.

## Supporting Information

Figure S1 **Overexpression of active ezrin or activation of endogenous ezrin induces actin-rich surface protrusions. A.** C2C12 myoblasts cultured on glass cover slips were transfected with GFP-fused wild type ezrin (Ez-WT) or its mutated variants (Ez-T567D and Ez-T567A) or GFP alone. After 24 h were fixed and stained with TRITC-phalloidin to decorate actin cytoskeleton and counterstained with DAPI to visualize nuclei (blue), and further imaged by CLSM. C2C12 cells were treated with the toxin C3-transferase, inhibitor of Rho, **B**, or with LPA, inductor of the Rho signaling pathway, **C,** for 2 min and then fixed and stained with TRITC-phalloidin and counterstained with DAPI (blue) to visualize actin cytoskeleton and nuclei respectively.
(TIF)

Figure S2 **Purity controls for the cytoplasmic and nuclear fractions.** Cytoplasmic and nuclear extracts were obtained from C2C12 cells as described in Material and methods, and further subjected to Western analysis using antibodies against Sp3 and lamin B1 or against GAPDH and Calnexin (Clnx), to prove the purity of cytoplasmic and nuclear extracts respectively.
(TIF)
