# Supplementary figures and images for: Nuclear Import of β-Dystroglycan Is Facilitated by Ezrin-Mediated Cytoskeleton Reorganization
Source: PLoS One. 2014 Mar 5;9(3):e90629. doi: 10.1371/journal.pone.0090629 (PMC3944073; doi:10.1371/journal.pone.0090629)

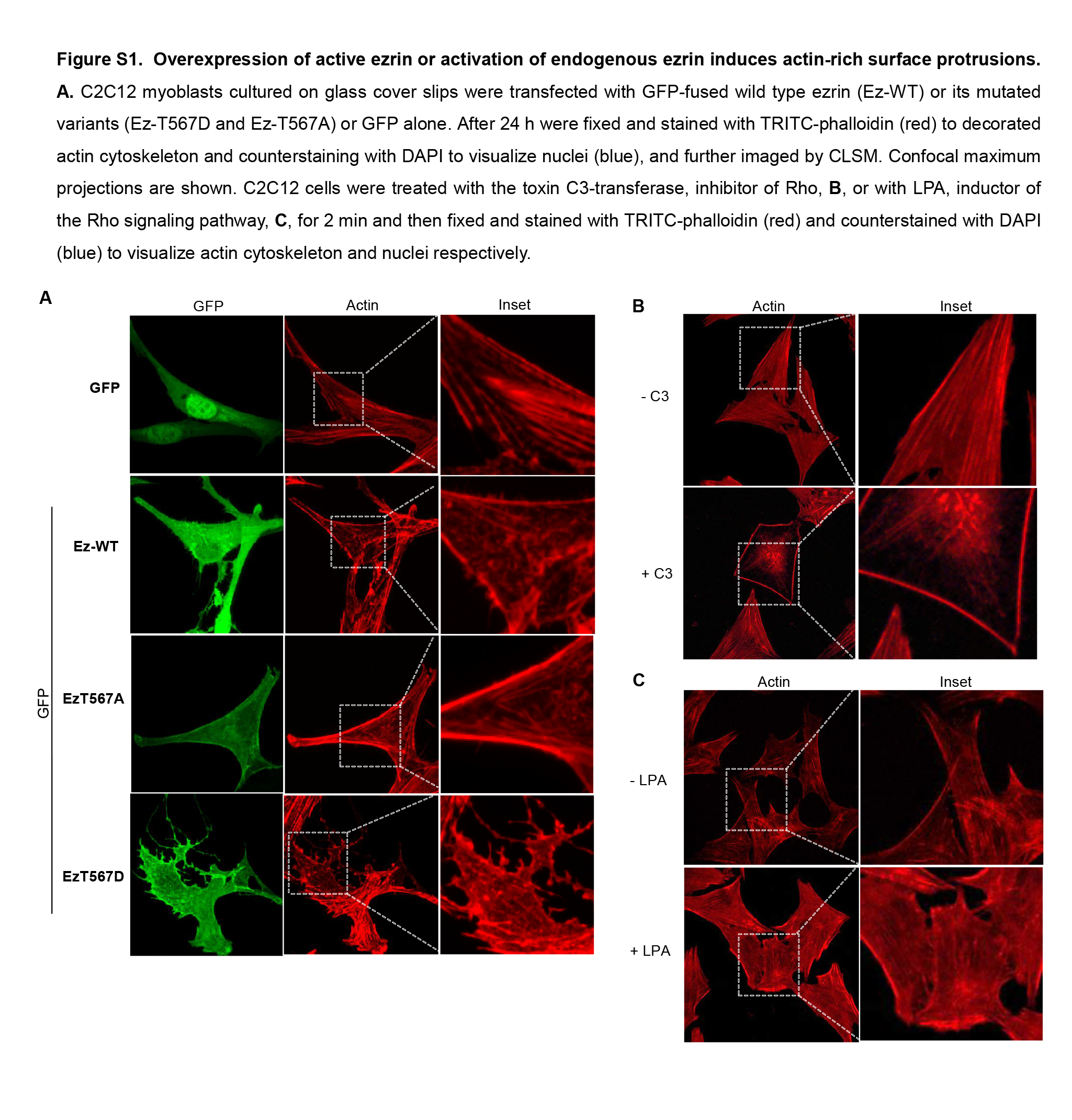

Supplement: Figure S1 — Overexpression of active ezrin or activation of endogenous ezrin induces actin-rich surface protrusions. A. C2C12 myoblasts cultured on glass cover slips were transfected with GFP-fused wild type ezrin (Ez-WT) or its mutated variants (Ez-T567D and Ez-T567A) or GFP alone. After 24 h were fixed and stained with TRITC-phalloidin to decorate actin cytoskeleton and counterstained with DAPI to visualize nuclei (blue), and further imaged by CLSM. C2C12 cells were treated with the toxin C3-transferase, inhibitor of Rho, B, or with LPA, inductor of the Rho signaling pathway, C, for 2 min and then fixed and stained with TRITC-phalloidin and counterstained with DAPI (blue) to visualize actin cytoskeleton and nuclei respectively. (TIF) [file pone.0090629.s001.tif]

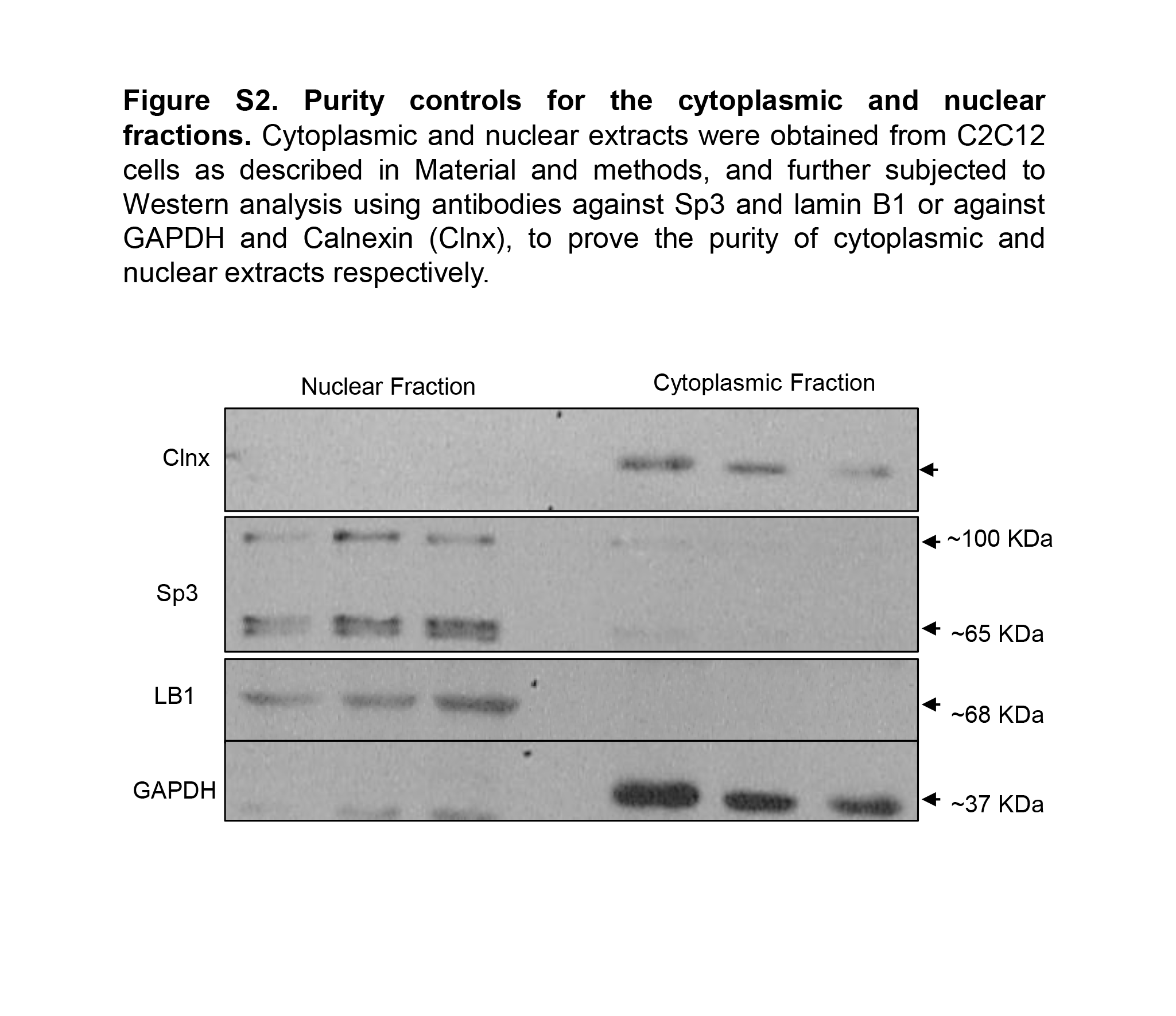

Supplement: Figure S2 — Purity controls for the cytoplasmic and nuclear fractions. Cytoplasmic and nuclear extracts were obtained from C2C12 cells as described in Material and methods, and further subjected to Western analysis using antibodies against Sp3 and lamin B1 or against GAPDH and Calnexin (Clnx), to prove the purity of cytoplasmic and nuclear extracts respectively. (TIF) [file pone.0090629.s002.tif]
